# Design of a deep learning prediction model for Alzheimer's and Parkinson's Disease using MRI images

**DOI:** 10.3389/frai.2026.1777236

**Published:** 2026-04-09

**Authors:** Velu K., N. Jaisankar

**Affiliations:** School of Computer Science and Engineering (SCOPE), Vellore Institute of Technology (VIT), Vellore, Tamil Nadu, India

**Keywords:** Alzheimer's disease, deep learning, feature extraction, Grad-CAM++, magnetic resonance images, optimization, Parkinson's disease

## Abstract

**Introduction:**

Alzheimer's disease (AD) and Parkinson's disease (PD) are types of neurodegenerative diseases that affect the body and get worse over time. The cause of AD mainly involves the buildup of protein which are abnormal, issues with the immune reaction, death of neurons. Different from this, the death of the neurons that make dopamine leads to PD and causes both motor and non-motor problems. MRI images are used to provide an early and correct diagnosis to enable timely treatment planning and management of the disease.

**Methods:**

In this paper, a design of an AI-based deep learning framework is proposed for the classification of neurodegenerative disease based on the brain MRI data. The pipeline that we propose begins with data preparation including data augmentation using InceptionGAN for augmentation of the dataset and fixing of class imbalance issues. A composite method of feature extraction using ConvNeXt and MaxViT along with the Cross-Fusion Attention model, worked well to capture local and global spatial features. Bayesian Optimization and Genetic Algorithm are used to optimize hyperparameters for improving the performance of the model.

**Results:**

The Hybrid Deep Neural Network (HDNN) is the last classifier with an accuracy of 97.4%. Based on performance accuracy, F1-score, the model is strong and reliable. We used Gradient-weighted Class Activation Mapping++ to explain how regions of interest in the brain influence our model's decisions.

**Discussion:**

This study offers an interpretable and high-performing deep learning framework for the early and precise prediction of neurodegenerative disorders utilizing MRI imaging, thereby enhancing clinical decision-making and patient care.

## 1 Introduction

Brain diseases are a diverse category of maladies affecting the brain. These maladies include neurodegenerative diseases [Alzheimer's disease(AD), Parkinson's disease(PD)], vascular diseases (stroke), infections (meningitis and encephalitis) and physical injuries (concussion). These conditions can impact cognitive function, movement, mood, and language, and can be caused by genetic factors, injuries, infections, or degeneration of nerve cells. Alzheimer's disease is a condition that causes a significant decrease in skills such as social skills, thinking, and behavior, and it leads to brain shrinkage and cell death in the brain. Memory loss is one of the most prominent signs of Alzheimer's, which is the most common cause of dementia. The symptoms of the disease worsen over a period of time, starting with trouble recalling recent conversations and events. As Alzheimer's disease gets worse, it alters the brain in ways that can make it harder to think, make decisions, plan, modify behavior, and remember things. Parkinson's disease affects elements of the body controlled by nerves, especially the neurological system. It is characterized by tremors and symptoms that start slowly. Even though there is no cure for Parkinson's disease, medications and, if needed, different types of surgery can help control its symptoms. The basic symptoms, which might show up in different ways in different people, are tremors, changes in speech, alterations in handwriting, postural instability, and slowness of movement (Better, 2023; Parkinson's Foundation, 2022).

Early detection of Alzheimer's and Parkinson's diseases can improve management and therapy of these neurological disorders. Through the analysis of vast volumes of data from multiple sources, Artificial Intelligence (AI) can be extremely helpful in the detection of these diseases. incorporating genetic data, brain scans, medical records, and other pertinent information (Schork, 2019; Haleem et al., 2019). The amount of brain shrinkage seen in the hippocampus and in the temporal lobes gets worse as Alzheimer's disease progresses. Longitudinal studies that use Magnetic Resonance Imaging (MRI) scans make it easier to find people who are likely to get the condition over time. Neuroimaging techniques have come a long way in recent years, and this has led to the generation of large, multimodal datasets in the field. A lot of research has shown that MRI is a common imaging technology utilized in clinical practice. MRI scans of adult brains can correctly predict AD (Noor et al., 2020). The potential of AI to enhance early detection, enable better treatment and management, and quicken the development of novel medications and treatments makes it crucial for identifying Parkinson's and Alzheimer's disorders. In addition, early diagnosis and health care systems for Parkinson's and Alzheimer's disease are huge problems. Early detection of the disease may result in a decreased number of hospital admissions and treatments. This may reduce overall healthcare expenditure (Joshi et al., 2004). AI and its advancements in identifying Alzheimer's and Parkinson's diseases could completely revolutionize how we detect and treat these diseases. AI can improve the lives of millions of people suffering from these diseases through increasing accuracy, helping in early detection, and increasing the pace of research. As a result, the studies in the literature are many to use deep learning models to automatically detect different disorders (Laske et al., 2006). Chen et al. (2024) identified a challenge that is inherent to the use of multipool CEST MRI for diagnosing PD due to the time-consuming process of collecting complete Z-spectra. The authors created a new method using deep learning techniques to combine many of these techniques into one framework for the collection, reconstruction and analysis of under-sampled Z-spectra that retains clinically relevant multi-pool diagnostics. However, while elegant and motivated by clinical needs, this approach places significant dependence upon the spectral priors that are learned through deep learning and thus is sensitive to differences among field strengths among sites. Therefore, interpretability remains unanswered.

In an AI-Driven Framework for Early Detection of Neurological Disorders, Abiodun et al. (2025) used both brain MRI and CT images with EfficientNetB0, with Alzheimer's disease (AD), Parkinson's disease (PD) and healthy controls as their target, achieving a total of 95% overall accuracy, demonstrating that lightweight convolutional neural networks (CNNs) can be applied clinically in low-resource countries. There is no provision to explore complex global patterns, which can occur in such 2D images. Also, there is no concept explanation provided to ensure integrated explainability. A classical ML paradigm has been introduced by Singh et al. (2019) focusing on multiclass neurodegenerative disease diagnosis through neuroimaging on a large pool of 2,540 participants with AD, PD, MCI, SWEDD, and healthy controls. They obtain a classification accuracy of 87.9% with the help of PCA, Fisher discriminant ratio, and LS-SVM. Although the work on multiclass classification helped in stepping beyond binary classification problems, the need remains for the development of complex brain patterns through deep learning models.

Katkam et al. (2025) proposed a deep learning model that provides a way to classify neurodegenerative diseases (NDDs) into multiclass based on MRI images, utilizing feature extraction from CapsNet, modifying DenseNet169 to address classification and employing Enhanced DeepLabV3+ to segment regions within the image. In addition, the interpretability of their model can only be implied through segmentation; therefore, further work needs to be done on creating hybrid architecture models that utilize Attention-based XAI techniques to provide more explicit explanations of the model's decision-making process. Goyal et al. (2025) proposed a multi-class classification approach using deep learning for the differentiation of neurodegenerative disorders such as AD, PD, and normal controls from magnetic resonance imaging. Their work is effective in the classification of the aforementioned disorders using CNN-based feature learning and traditional optimization techniques. But the method is limited, as it is mainly dependent on single-stream and minimal attention views, which can neglect the holistic spatial connections in the human brain. Hence, we propose a deep learning model for neurodegenerative disease prediction to address these research gaps.

The main contributions of the study are as follows:

• Developed an AI-driven deep learning framework for classifying AD, PD, and Healthy Controls using MRI data.

• Introduced a hybrid deep learning model combining ConvNeXt and MaxViT with Cross-Fusion Attention for robust MRI feature extraction.

• Optimized model performance using Bayesian Optimization and Genetic Algorithm for effective hyperparameter tuning.

• Applied Inception Generative adversarial network (GAN) for advanced data augmentation to handle class imbalance and enhance dataset diversity.

• Integrated Gradient-weighted Class Activation Mapping Plus Plus (Grad-CAM++) for explainable AI, enabling visualization of critical brain regions in disease classification.

The subsequent structure of the research is arranged as follows: The Related Works section examines the current literature on the detection of neurodegenerative diseases with MRI and deep learning techniques. The Materials and Methods section states the proposed framework, including data preprocessing, hybrid feature extraction, and optimization techniques. The investigational findings and performance assessment of the model are discussed in the results section. The conclusion sum up the main results and highlights prospective avenues for study.

## 2 Related works

This section highlights the established features of Alzheimer's disease (AD) and Parkinson's disease (PD), recognized as separate neurodegenerative conditions with distinctive clinical and pathological features. It introduces the concept that these diseases form part of a spectrum of neurodegenerative diseases, which is often complicated by overlapping or coincident pathologies. This provides the rationale for a unified approach to their stratification.

Alrawis et al. (2025) presented an FCN-PD model in 2025, incorporating EfficientNet and attention modules for extracting local and global features of MRI images in predicting Parkinson's disease. The model attained a highest accuracy of 97.2% on the PPMI dataset, proving high discriminative ability. Yet, the research is limited to PD, employs a solitary feature extraction backbone, and does not emphasize explainability analysis. Acikgoz et al. (2025) proposed an attention-enhanced residual dense CNN model to support classification in PD patients using MRI. They have achieved 94.44% accuracy. The significance of reusing dense features, attention, or both is demonstrated by this study. The drawback within this model is that it supports binary classification, offline data augmentation but ignores class imbalance while using the concept of generative models. Hussain et al. (2025) proposed a CNN-Swin Transformer fusion approach to efficiently explore both texture information and long-range relationships of MRI data with a maximum accuracy of 97% on PPMI. This paper clearly verifies that there is a necessity for hybrids based on CNN & Transformer architecture. However, there is no exploration of hyperparameters, as well as explanation techniques for feature fusion. Cao and Long (2025) have developed a multimodal RACF fusion system using SNP genetics and MRI information via transformer fusion, achieving accuracies of 91.2% and 0.94 AUC, respectively, indicating their success in this area. The downside to this approach is that genetic data increases the processing time, thereby limiting how scalable it could be in a real clinical environment.

Shawa et al. (2025) used a data-driven model of disease progression on a big data MRI dataset, establishing atrophy subtypes in patients with Parkinson's disease, gaining insight into disease variability. However, this model is unsupervised and not meant for patient-level diagnosis or classification. Mmadumbu et al. (2025) conducted an experiment to detect early Alzheimer's using two separate pipelines for clinical data and MRI using deep learning. Their experiment using LSTM-FNN for clinical data and ResNet50-MobileNetV2 for MRI yielded an accuracy of 96.19% on the ADNI set and 99.82% on the NACC set. It becomes quite effective when the data sources are distinct. The drawback lies in the fact that the networks are trained individually. There is no spatial explanation for the outcomes obtained from MRI. Pradhan et al. (2024) concentrated on Alzheimer's disease detection via MRI images using deep CNN models like CNN, DenseNet, ResNet50, and VGG. The results were better, especially when feature selection was considered, thus supporting the use of features learned from deep models for AD diagnosis. The model relies too much on conventional CNN models and manual removal of features. The model also lacks attention mechanisms and interpretability techniques. A transfer learning model using EfficientNetB7 for multi-class classification in Alzheimer's disease based on MRI images was proposed by Suchitra et al. (2025) On testing the model using the ADNI database, the model obtained an accuracy measure of 98.2%. While the accuracy values are remarkable, in fact outstanding, the paper explores only one kind of model for the backbone architecture. Also, it does not reveal any information about the interactions pertaining to the different models' cross-feature variables or any information about the models' decision-making.

Mousavi et al. (2025) studied a large dataset of more than 6700 MRI brain scans pulled together from an extensive primary study on 'an MRI Study Evaluating Brain Imaging and Diagnostic Performance or Efficacy of VGG16, VGG19, Xception, and InceptionResNetV2 Model's in Classifying Alzheimer's Disease Patients'. The findings of this study indicated that the InceptionResNetV2 Model could classify all stages of the Alzheimer Disease Patient nearly flawlessly. However, the purpose of the study was not to develop a new model architecture but rather to compare the four previous models to determine which model performed better. (Güven 2024) conducted a review of research on the effectiveness of transformer architectures such as the Swin Transformer, ViT and BEiT in classifying MRI images of patients diagnosed with Alzheimer's Disease and Parkinson's disease. The authors have been able to report accuracy above 80% with a balanced dataset, proving that transformers have potential in diagnosing neurodegenerative disorders. However, the smaller dataset used and lacks hybrid models or optimized fusion models.

Alhudhaif (2024) work used a combination of methods to improve the quality of MRI images. First, they used a very deep super-resolution network to find deep-level features, and then they used a random subspace approach via KNN to find those features. The achievement of 99.11% accuracy on three-class classification of AD, PD, and normal samples is certainly remarkable. However, this method is heavily dependent on traditional ensemble approaches and provides negligible information on where this network is scanning inside the brain. A multi-stage model called the EfficientNet was proposed by Shastry et al. (2025) aiming for both Alzheimer's and Parkinson's disease detection in MRI images. In this model, the better performer was always the EfficientNet-B6 model, which boosted validation accuracy by at most 6.5% for the multi-class datasets. Although its idea is carefully crafted and generalized well, this model still uses only one backbone model support. Asaduzzaman et al. (2025) introduced ALZENET in 2025 as a combination of VGG16, Inception-V3, and ResNet50 with SMOTE to overcome class imbalance when dealing with Alzheimer's MRI images. They achieved 97.31% accuracy for all four stages of dementia and a web-based tool for diagnosis. Though successful in prediction, the model is only focused on AD patients and uses CNN features. Lack of transformer-based global reasoning and lack of explanations. Lu et al. (2025) provided a paradigm for understanding Parkinson's disease diagnosis using a combination of clinical scores, voice analysis, and genetic factors obtained via stacked ensemble learning along with SHAP analysis. The approach obtained AUC scores higher than 0.96, which is interpretable. But this research purposely does not include neuroimaging and combines the various data sources at the model level, and not at the feature level.

Based on the literature, relevant studies investigating the development of MRI-based Classification for Alzheimer's Disease and Parkinson's Disease using Convolutional Neural Networks (CNN), Transformers, Hybrid Deep Learning Models, and Multimodal Approaches indicate several major limitations that persist across all studies. The majority of studies originate from datasets that are limited, imbalanced, resulting in limited generalizability of results. The majority of studies utilize basic augmentation techniques for images that do not sufficiently address class imbalances or expand the variety of the dataset. Furthermore, the majority of studies only utilize either CNN or Transformers, but not both; this makes a struggle between locally capturing structural attributes and capturing global contextual features. Furthermore, a majority of studies lack hyperparameter tuning strategies; consequently, the resulting architectures are typically suboptimal or excessively computationally intensive. The lack of Explainable AI (XAI) techniques makes the models decision process remain opaque, thus limiting the clinical trust placed upon the model. To address these concerns, the proposed work employs the Inception-GAN for advanced augmentation for the introduction of additional datasets, employs a ConvNeXt-MaxViT hybrid with Cross-Fusion Attention for feature extraction improvement, applies Bayesian Optimization and Genetic Algorithm to optimally tune hyperparameters resulting in a highly accurate (97.4%) HDNN classifier, and implements Grad-CAM++ to provide a transparent and definitely clinically interpretable prediction.

## 3 Materials and methods

**Figure 1:**
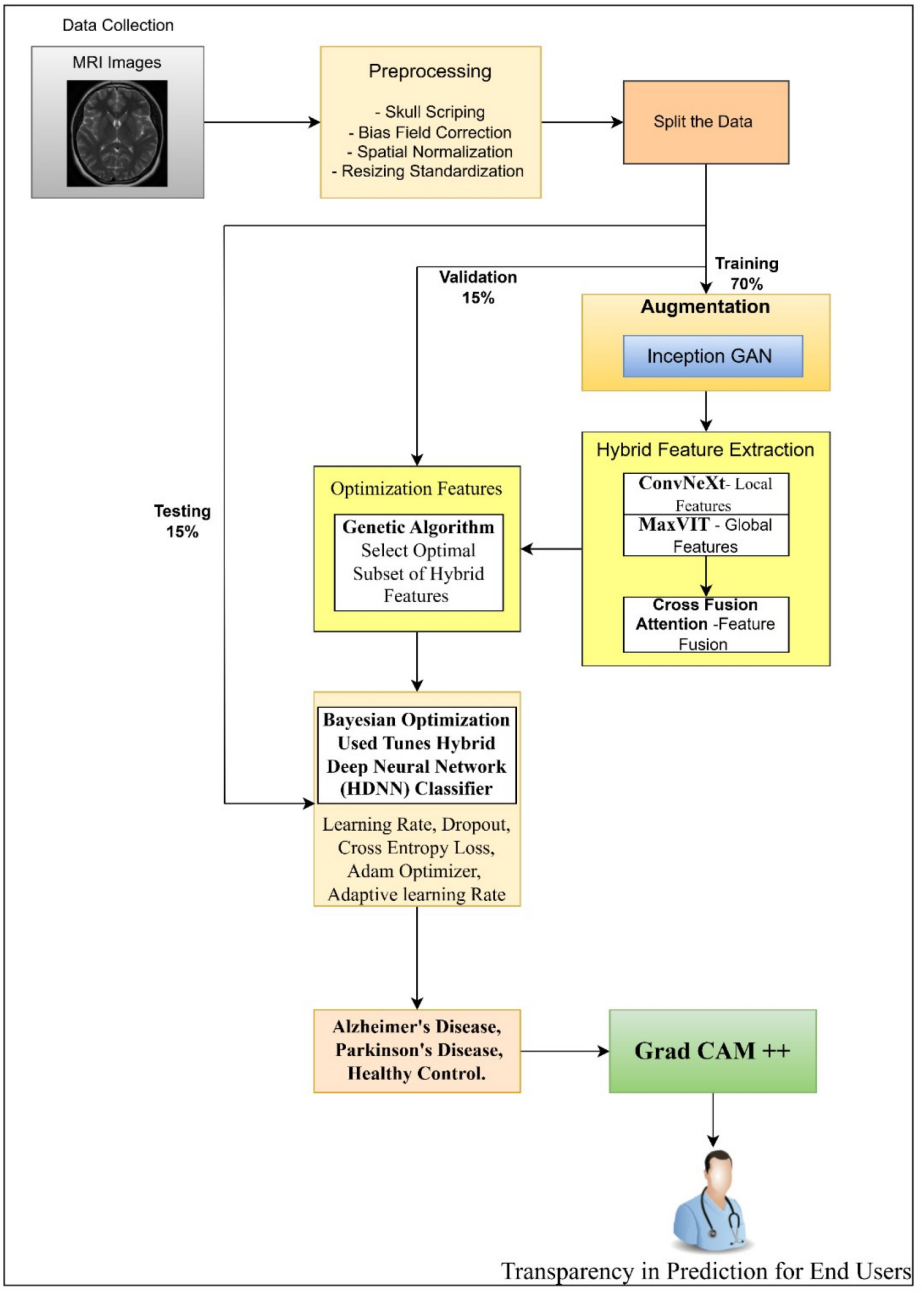
overview of the proposed model.

This part describes the structured approach used to categorize various brain disorder groups based on MRI scans. In the methods part, advanced steps were used before analysis methods, extensive data expansion, identifying key traits, adaptive tuning of includes features and a hybrid deep learning model for classification. Each subsection is organized to improve precision, lower overfitting risk, enhance feature generalization, also make outcomes easier to understand. An overview of the full pipeline appears in the proposed system layout, as shown in Figure 1.

### 3.1 Dataset description

The model starts by gathering high-resolution T1-weighted Magnetic Resonance Image scans are act as the main tool for detecting brain degeneration. Since these images are from open-access standard databases, like the Alzheimer's and Parkinson's dataset (Samanta, 2023). they ensure consistency across analyses. Instead of relying on custom data, pre-existing MRI volumes form the core input needed to classify Alzheimer's (AD), Parkinson's (PD), and healthy individuals (HC), as outlined in Table 1. MRI detects subtle brain structure changes; therefore, it provides solid data for computing tasks while acting as the first step in preprocessing.

**Table 1:** Dataset description.

Attribute	Field	Description
Dataset Info	Dataset name	Alzheimer's and Parkinson's disease 3-Class dataset
		Source / platform	Kaggle
	Classes / labels	CONTROL, AD, PD
	Image type	Brain MRI / Medical Images
	Number of classes	3
Class details	CONTROL	Healthy individuals with no neurological disorders
	AD	Patients diagnosed with Alzheimer's disease
	PD	Patients diagnosed with Parkinson's disease

### 3.2 Data preprocessing

**Figure 2:**
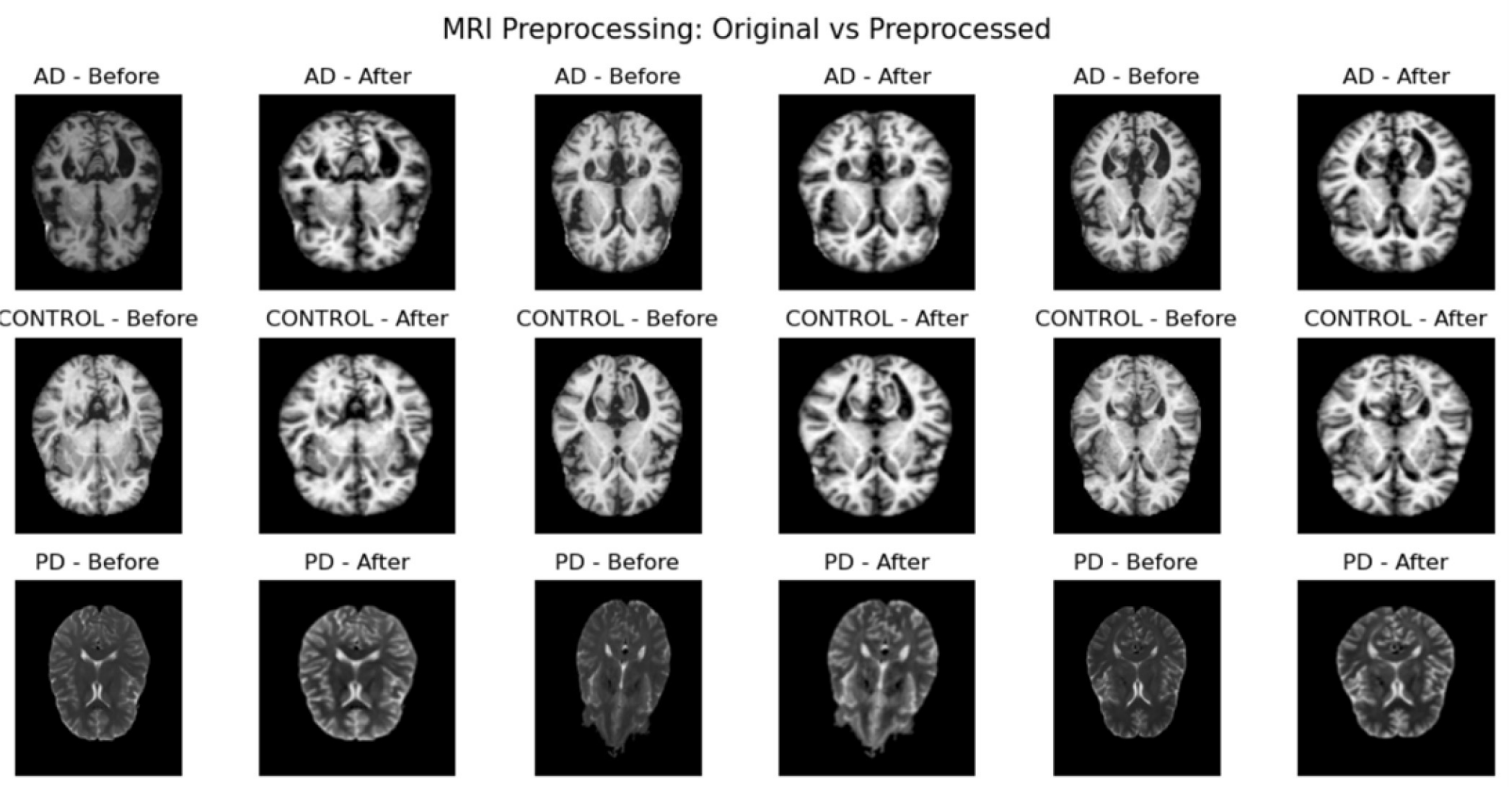
Preprocessed samples.

Each MRI volume was subjected to a set of preprocessing steps to ensure better structures and more consistent features. The skull stripping is applied first to remove the tissues that are non-brain tissues, resulting in a clean representation of the respective anatomy of the brain. Next, we apply a bias field correction, which reduces smooth intensity distortions caused by scanner irregularities. The images are then spatially normalized to a common anatomical template to achieve voxel-wise correspondence across subjects. In the end, size reduction and statistical intensity normalization make all inputs geometrically and numerically consistent, as shown in Figure 2. The sequence of pre-processing standardizes the dataset so that the downstream models will work on harmonized imaging inputs. After preprocessing, the dataset is split into training, validation, and testing sets based on a stratified distribution. Dataset allocation shares 70% for training, 15% for validation, and 15% for testing. This proportional split sustains class balance across all subsets, preventing bias during model building. The validation set is used to tune the hyperparameters and the test set is untouched until the very end performance evaluation.

### 3.3 Augmentation

**Figure 3:**
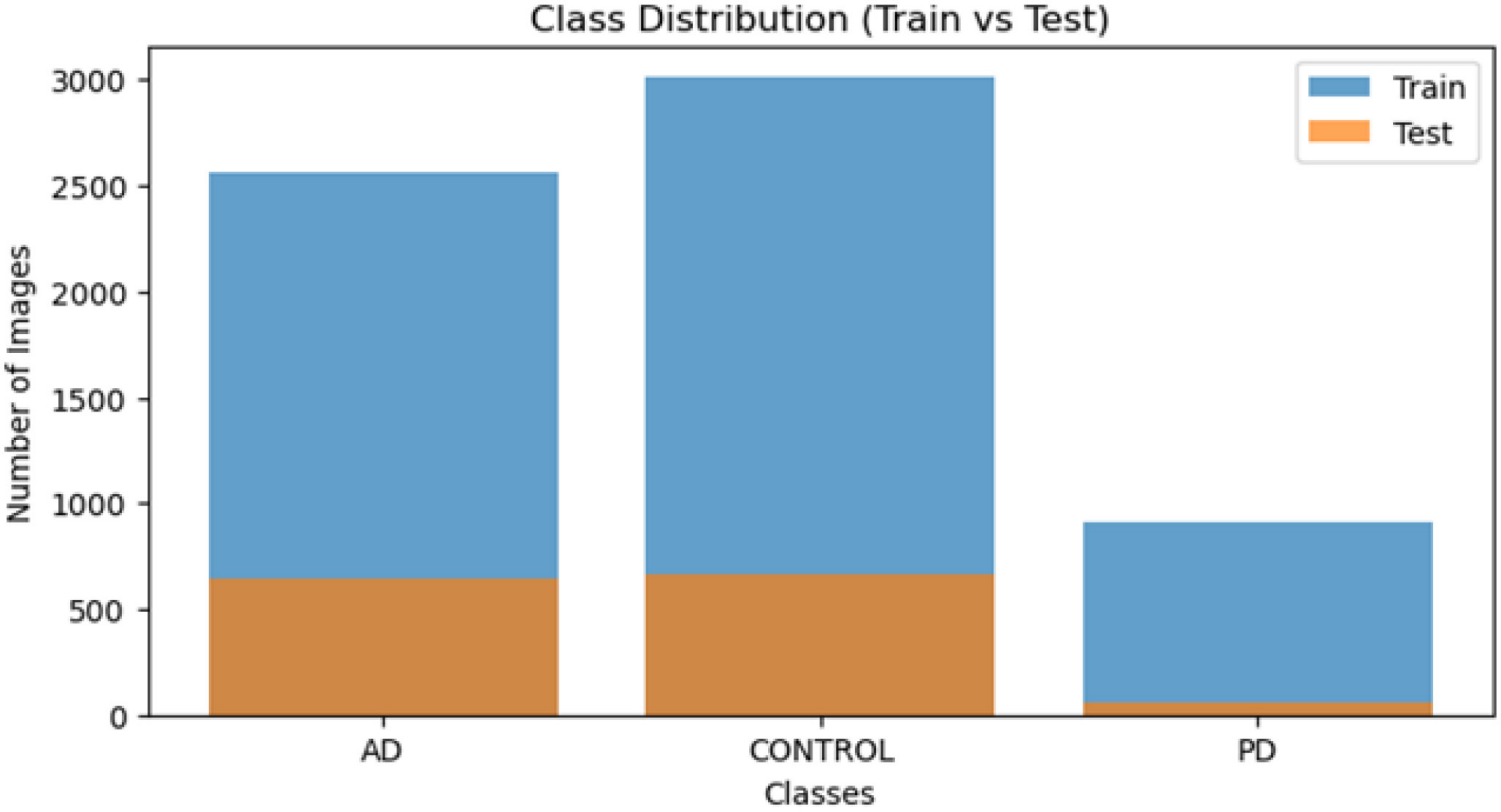
Class distribution of Alzheimer's, Parkinson's and Healthy Controls based on the training and testing set.

The Figure 3 shows that each of the three categories (AD, Control, PD) has its own unique spread of images throughout both the training set and the testing set. There is an imbalance in the images for each category; Although the Control category has the largest number of images, the PD category has the smallest number of images.

Class distribution reflects that the imbalance in the number of images for each category will affect the model's ability to correctly classify instances of PD. Therefore, to employ augmentation techniques and/or different ways of weighting the images by class during the training period, to ensure that all categories are treated equally in the final classification process. An Inception-based Generative Adversarial Network is used to handle dataset inequity and to further augment the variety of brain structural patterns that are seen during training. This augmentation framework generates realistic MRI-like images by learning from the statistical and anatomical distribution of the original scans. These synthetic images introduce verisimilar structural features to the dataset, exhibiting subtle variations in appearance with regard to their texture and spatial composition. Finally, these synthetic samples are combined with original ones in order to expand the diversity of the training set and reduce the chances of overfitting.

Unlike traditional GAN models, which have fixed Receptive Fields and are frequently unable to adequately represent intricate anatomical structures, the proposed inception GAN model includes a multi-scale Inception module in its generator design, which enables multiple levels of detail to be extracted simultaneously (e.g., fine textures, mid-level tissue structure, and large-scale brain morphology). The generator will take in a latent noise vector and then perform transposed convolutions to produce its output, where the main Inception block performs its functions as described. The key components of the main Inception block include parallel 1 × 1, 3 × 3, and 5 × 5 convolutions, and the Max-Pool branch for extracting all possible pathological cues (e.g., cortical thinning or ventricular enlargement, etc.). The discriminator is designed as a progressively deep Convolutional Neural Network (CNN) with spectral normalization to enforce anatomical correctness while penalizing all types of artifacts that would otherwise confuse the CNN and therefore guide the generator in creating high-quality synthetic MRI slice engineering. Both the generator and discriminator will be designed as class-conditioned GANs so that the AUGM can preserve the AD (Alzheimer's Disease), PD (Parkinson's Disease) and HC (Healthy Control) labels for all augmentations, with a feature-matching loss calculated to improve the GAN's training stability. The Inception GAN allowed for the expansion of the dataset by virtue of augmenting the existing data with subtle but clinically significant variations and, therefore, significantly reduced the rate of overfitting and increased the generalization potential of the hybrid CNN and Transformer Classifier.

The steps of the experimental pipeline were isolated from each other to ensure that they did not leak any information. Before the preprocessing, augmentation, or normalization steps, the dataset was first split into stratified training (70%), validation (15%), and test (15%) subsets at the subject level. The model training, hyperparameter tuning, GAN training, and feature optimization did not use any validation or test set samples. To prevent any form of data leakage, the stratified split into Dtrain, Dval, and Dtest is performed before any learning-based transformation. The Inception-GAN is trained exclusively on Dtrain, and all generated synthetic samples are appended only to the training set (Dfinal_train = Dtrain union Daug), while validation and test sets remain strictly real and untouched, consistent with the end-to-end procedure in Algorithm 3. In addition, intensity normalization (mean/standard deviation) is computed using training data statistics only, and the same parameters are applied to Dval and Dtest. Therefore, neither GAN generation nor preprocessing parameters are ever fitted using validation/test information, ensuring unbiased evaluation.

### 3.4 Hybrid feature extraction architecture

The augmented dataset undergoes processing through a dual-branch feature extraction system that combines the strengths of convolutional and transformer-based approaches. The ConvNeXt pathway focuses on capturing spatial features and detecting complex patterns within cortical and subcortical regions. Conversely, the MaxViT pathway utilizes self-attention mechanisms to illustrate the global anatomy and long-distance interactions. We apply a fusion attention via a gated mechanism on the extracted features from the backbone neural networks. This combination leads to a broad hybrid feature representation that encompasses fine-grained details as well as coarse-grained context information. The suggested framework includes the dual-level feature extraction process for brain MRI scans' global structural patterns as well as fine-grained anatomical details.

**Figure 4:**
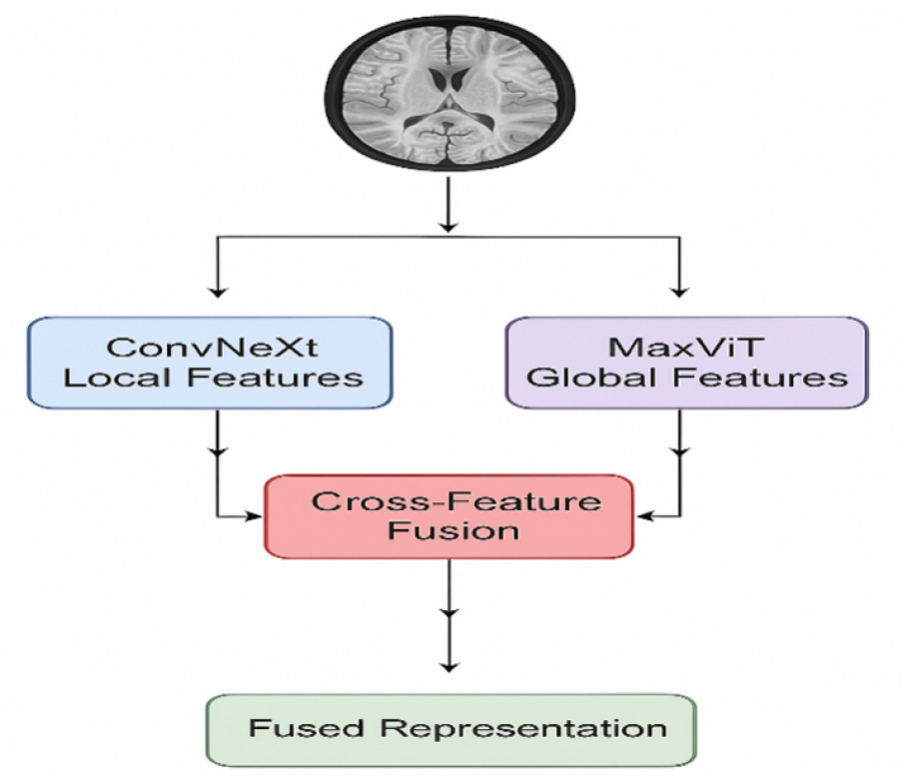
Dual path feature extraction.

To this end, a dual-path encoder strategy is followed that integrates the use of a ConvNeXt-inspired CNN to extract the local features and a MaxViT Transformer for the modeling of global features, as shown in Figure 4. The ConvNeXt-like encoder focuses on subtle, region-specific variations in cortical thickness, gray matter differences, and subcortical textures through depthwise convolutions, residual connections, layer normalization, GELU activation, and global average pooling to produce compact, informative feature vectors (Jin^1^ et al., 2022). Along with the local pathway, the MaxViT-based global encoder utilizes the expressiveness of local and global attention mechanisms to capture long-range dependencies and inter-regional relationships, potentially letting the model learn holistic brain structures, hemispheric asymmetries, and clinically relevant global patterns (Aslan et al., 2025). To fully exploit the discrimination power of both local and global features, a cross-fusion attention module first concatenates the feature vectors from both pathways and then computes adaptive importance weights, yielding a highly discriminative fused representation emphasizing the most representative features for classification. This integrated method improves the model's ability to detect minor brain degeneration while supporting broader applicability and minimizing overfitting - enabling effective feature tuning via GA alongside HDNN classification, which plays a key role in reaching high precision in this study.

### 3.5 Genetic algorithm for feature selection

To enhance hybrid features and enhance efficiency, a two-phase optimization approach is employed. A Genetic Algorithm to search over the feature space, and select the most useful subset by repeatedly applying selection and mutation (Díaz-Álvarez et al., 2022). This method eliminates features while keeping those with high discriminative power. Following feature refinement, Bayesian Optimization adjusts hyperparameters of the Hybrid Deep Neural Network, such as learning rate, dropout rate, and internal structural configurations.

The optimization phase is responsible for calibrating the feature space and model parameters to achieve an optimal performance level. This is illustrated in Algorithm 1.

Algorithm 1 Genetic algorithm for optimal feature selection. 
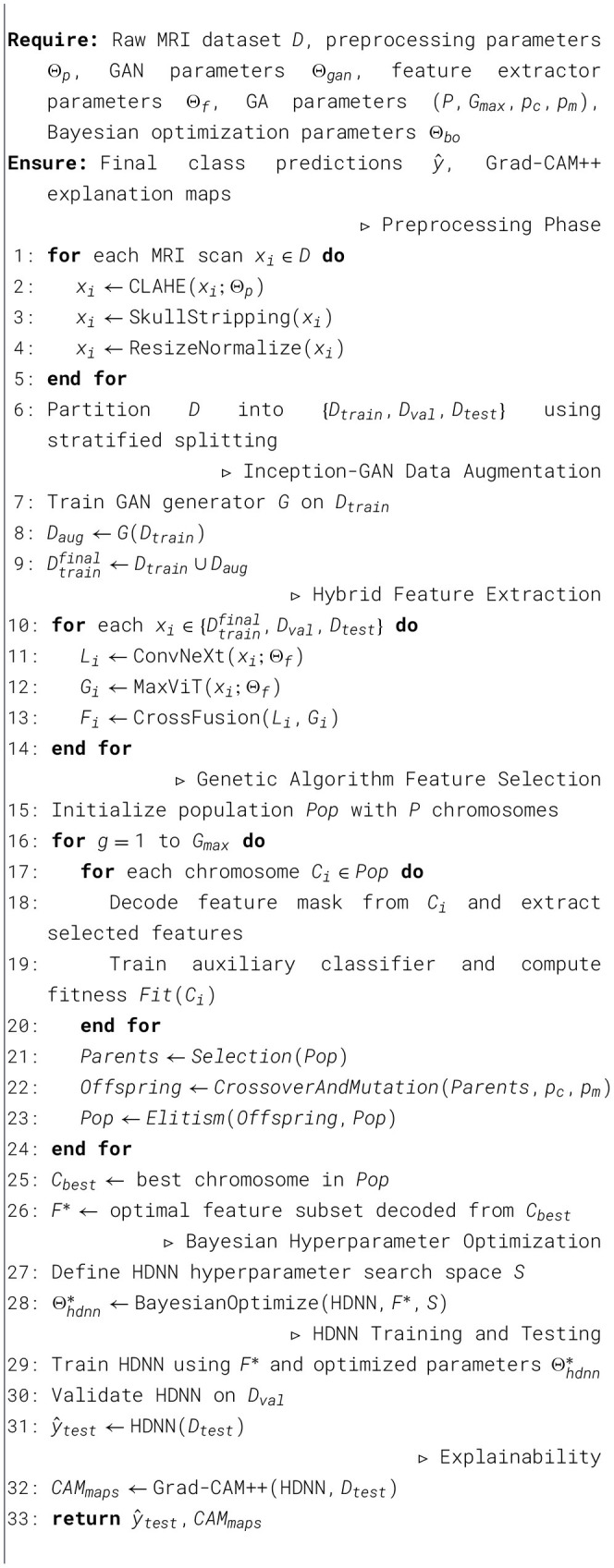

The complete configuration of the BO + GA hyperparameter optimization framework to enhance reproducibility and address the reviewer's concern. Our parameter search focused on the learning rate in the 1e-5 to 5e-4 range in log scale, the batch size of 8, 16 and 32, dropout rate between 0.2 and 0.6, weight decay in the 1e-6 to 1e-3 range and the type of optimizer (Adam/AdamW). The validation macro-F1 score was used as the optimization objective. The first Bayesian Optimization was used to explore the search region with ten random trials followed by more guided trials in the specific range. Thereafter, a Genetic Algorithm refinement was performed with a population size of 16, 8 generations, and a mutation rate of 0.02 using tournament selection and elitism. The convergence was either hitting the upper limit of generations or observing no improving validation in successive generation. Throughout our work, all optimization activities were performed solely on the training and validation sets, and the test set was not seen at all. Per optimization trial there was an average time duration of 15-20 minutes. This will guarantee methods transparency and full experimental reproducibility.

### 3.6 Hybrid deep neural network training

The hybrid neural network used as a classifier is trained by the refined feature vectors. In training cross-entropy loss function is used, while the Adam optimizer is used for updating. The network learns distinct differences between Alzheimer's, Parkinson's, and healthy control groups after various iterations. By using optimized features and adjusting hyperparameters, the model can converge effectively while ensuring prediction stability and generalisability, as shown in Table 2. The proposed pipeline involves the integration of local (ConvNeXt) and global (Transformer) feature extraction methods through Genetic Algorithm-based feature selection. This results in decent performance with 97.4% accuracy. This is possible by preserving the best discriminative information for classification purposes. Optimization of the HDNN classifier for hidden (Kaliappan et al., 2024), learning rate, dropout rate, and class weights is given in the Table 2.

**Table 2:** Hyperparameters of the proposed end-to-end pipeline.

Module	Critical hyperparameter	Value/range	Impact
Dataset split	Training/validation/test	70%/15%/15%	Stratified split ensuring balanced class representation
GAN (Lightweight)	Synthetic images per class	50	Improves data variability and robustness with minimal overhead
Feature Extraction	Local ConvNeXt filters	32 → 64	Captures fine-grained local spatial patterns
	Global Transformer layers	3	Learns long-range dependencies and global contextual features
	Embedding dimension/Attention heads	192/3	Enables efficient multi-head self-attention
GA Feature Selection	Population size/Generations	16/8	Selects compact and discriminative feature subsets
	Mutation rate	0.02	Prevents premature convergence during evolutionary search
Classifier (HDNN)	Hidden units	256	Optimized via Random Search and Bayesian Optimization
	Learning rate	1 × 10^−3^	Ensures stable and efficient training convergence
	Dropout rate	0.4	Reduces overfitting and improves generalization
	Class weighting	Balanced from labels	Mitigates class imbalance during model training
Training	Epochs	100	Early stopping with patience of 8 epochs
Calibration	Temperature scaling	0.5–5.0	Minimizes validation Negative Log-Likelihood (NLL)

It was created using inception GAN-based augmentation methods that introduce limited variability that aids in the generalization of the model. Training consisted of 100 epochs, including an EarlyStopping component for a maximum patience setting of eight, ensuring convergence prior to overfitting. Using temperature scaling for prediction confidence, all of the major hyperparameters support optimal learning and effective multi-classification of an input dataset. The proposed model that is put forward gets to a time complexity that is well-balanced by the use of a light CNN for local features together with a transformer for global context, thus maintaining the computation at a manageable level. Despite the transformer making the cost quadratic in the sequence length S, the optimized patching method significantly cuts down on this cost. The entire process continues to be computationally efficient, giving the benefit of quicker inference over the deep standalone Transformer models and at the same time surpassing the performance of the purely CNN-based models, as shown in the Table 3. Therefore, the proposed architecture can achieve better accuracy and moderate computational cost.

**Table 3:** Time complexity analysis of the proposed model.

Stage	Main operations	Simplified time complexity	Computational load
Preprocessing	Skull stripping, CLAHE, resizing, normalization	*O*(*n*^2^)	Low
GAN-Based Augmentation	Generator and discriminator forward passes	*O*(*N*·*A*)	Medium
ConvNeXt Feature Extraction	Multi-layer convolution operations	*O*(*n*^2^) per layer	High
Transformer Feature Extraction	Self-attention over patch tokens	*O*(*p*^2^)	Very High
Feature Fusion + Embedding	Linear projection and attention-based fusion	*O*(*F*)	Low
GA Feature Selection	Population evolution over generations	*O*(*P*·*G*)	Medium
Bayesian Optimization	Iterative hyperparameter tuning	*O*(*I*)	Low
HDNN Classification	Dense and attention-based inference	*O*(1)	Low
Grad-CAM++ Explainability	One forward and one backward pass	*O*(1)	Low

Bold values indicate the best performance among the compared methods/experiments.

### 3.7 Explainability through gradient-weighted class activation mapping plus plus (Grad-CAM++)

After classifying, the model's predictions are explained by Grad-CAM++. This visualization highlights the parts of the anatomy that are most important to the network's decisions. Heatmaps are generated for each outcome displaying the attention intensity in the pertained MRI slice. You can see the implementation in Algorithm 2. This interpretability layer is an important step to foster transparency as it allows clinicians and researchers to verify choices made by the model as well as the structural reasons behind the categorization choice made by the model (Chattopadhay et al., 2018).

Algorithm 2 Pseudocode for Grad CAM++ heatmap generation. 
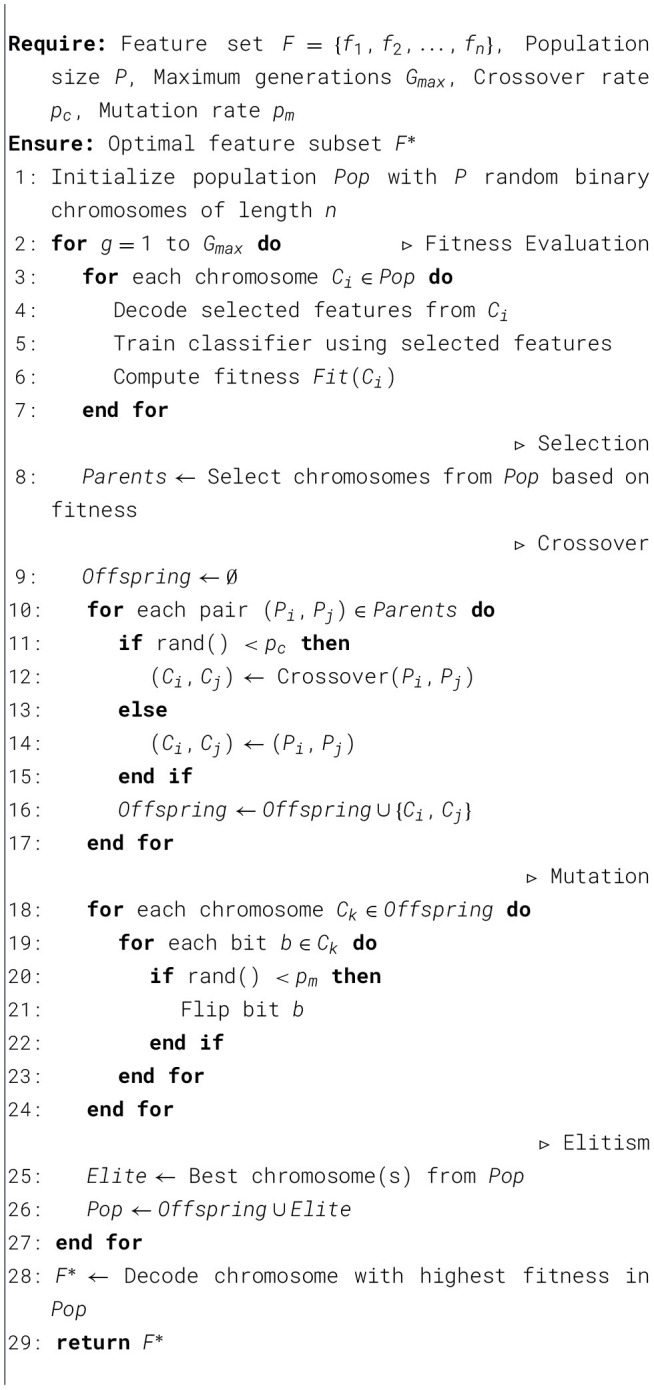

This interpretability layer is essential for promoting transparency, allowing clinicians and researchers to validate model choices and understand the structural signals influencing categorization results.

Combined, the proposed method provides an interconnected framework for automatic analysis of neurodegenerative disorders via MRI. By utilizing preprocessing, augmentation via GAN, combined feature extraction and optimization, the model identifies fine and large anatomical markers relevant to neurological diseases. The deep learning classifier in combination with an interpretability tool further reinforces the approach and culminates in a sustainable method that possesses accuracy, reliability and interpretability as shown in Algorithm 3. Thus, the methodology is a well-rounded methodology that can potentially contribute toward academic exploration and clinical decision-making in the neuroimaging-based diagnosis of neurological disorders.

Algorithm 3 End-to-end neurodegenerative disease classification model. 
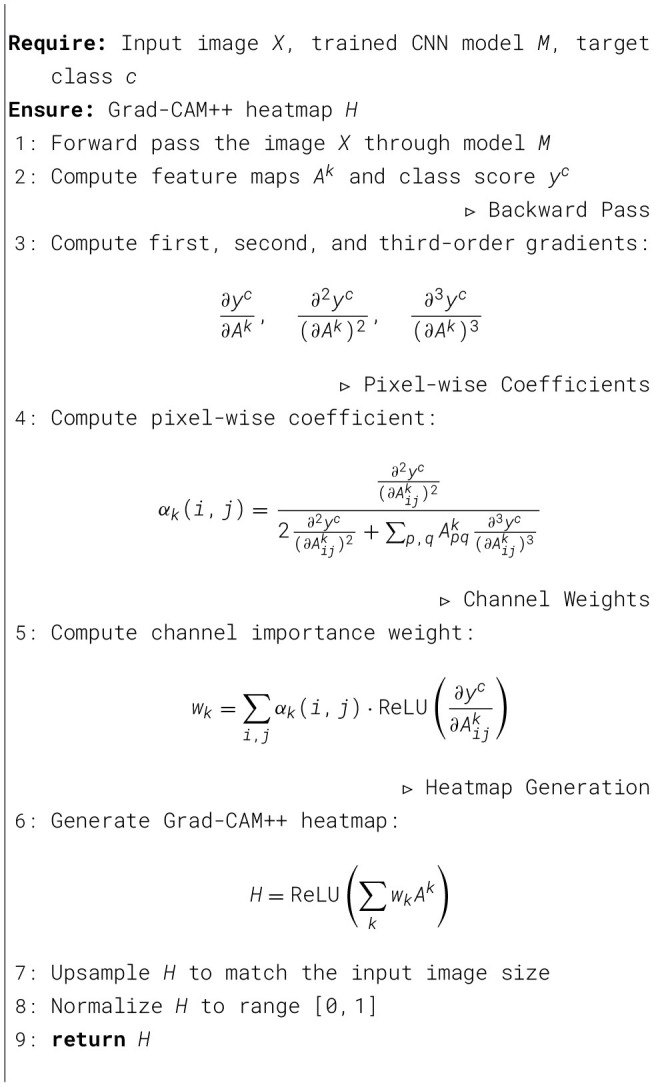

### 3.8 Model evaluation

This section essentially covers Alzheimer's Disease (AD) and Parkinson's Disease (PD), which can be considered significant as neurodegenerative ailments with progression mechanisms, as well as their influence on cognitive and motor functions. It also emphasizes the importance of early detection using advanced computational algorithms evaluated through performance metrics. Because of the class imbalance in the dataset, especially the fewer number of PD instances compared to other classes, overall accuracy may give a misleading impression of performance. For a robust evaluation, we also report macro-averaged metrics and balanced accuracy along with accuracy, precision, recall, and F1-score. Macro-averaging calculates the average of all classifications of all classes and lowers the effect of the larger number of classes. This ensures that more powerful classes do not impact the score disproportionately. Balanced accuracy is simply computed as the average of the recall values for all classes. Thus, it is the best metric to use for imbalanced datasets. It gives a more accurate assessment of classifier performance when underrepresented, such as in the case of PD.


(1) $$\begin{array}{l} \text{Accuracy}=\frac{\text{TP}+\text{TN}}{\text{TP}+\text{TN}+\text{FP}+\text{FN}} \end{array}$$

(2) $$\begin{array}{l} \text{Precision}=\frac{\text{TP}}{\text{TP}+\text{FP}} \end{array}$$

(3) $$\begin{array}{l} \text{Recall}=\frac{\text{TP}}{\text{TP}+\text{FN}} \end{array}$$

(4) $$\begin{array}{l} \text{F1 Score}=\frac{2\times \text{Precision}\times \text{Recall}}{\text{Precision}+\text{Recall}} \end{array}$$

Recall, as a metric, measures the model's ability to find positive instances about all actual positive data points correctly. The F1 Score serves as the harmonic mean of precision and recall. To summarize, accuracy, precision, recall, and the F1 score are essential measures for evaluating the effectiveness of a deep learning model.

## 4 Results and discussion

This section describes the results of the analysis from the multi-class classification experiments, which were conducted with brain scans for the prediction of Alzheimer's and Parkinson's diseases. All the experiments were performed on a high-performance computer composed of an Intel^®^ Core™ i7 at 3.0 GHz (8 core 16 thread) and NVIDIA^®^ RTX 4090 which has 24 GB of VRAM to quickly run deep learning experiments. This system was fully supported with 128 GB DDR5 RAM and 2TB NVMe SSD storage for quick availability of data that aid in handling large domains of MRI dataset. Ubuntu 22.04 LTS was the operating system. We implemented Python 3.10 for the programming environment.

In efforts to ensure robustness under class imbalance, macro-averaged precision, recall, F1-score, and balanced accuracy score were calculated. The model achieved similar high recall for PD class. This means that the classifiers are not biased toward the majority classes. The macro-F1 score was found to closely resemble (correlate with) the weighted F1 score. It suggests that there is stable generalization across all categories. Moreover, the balanced accuracy was found to be close to the overall accuracy, in other words. Thus, the performance is bona fide. The proposed framework successfully rectifies bias caused by sample imbalance and yields reliable detection of underrepresented PD samples.

**Figure 5:**
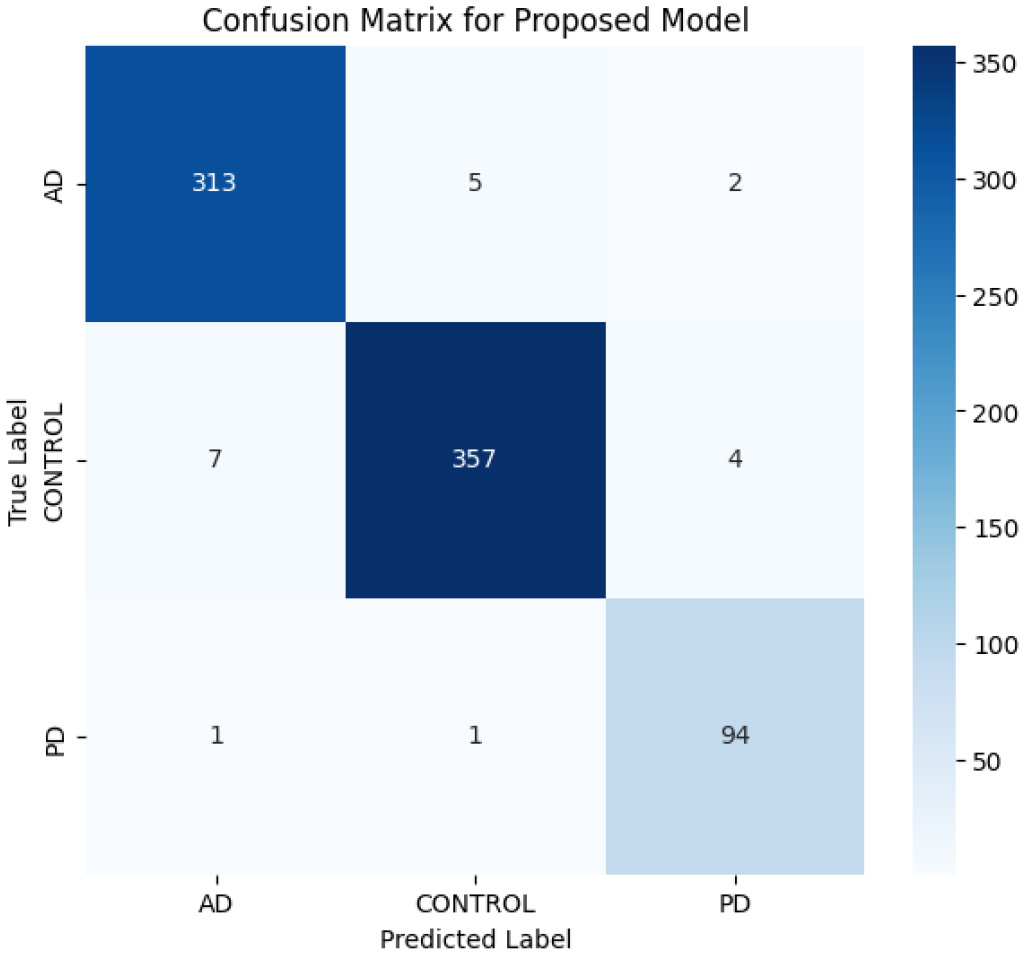
Confusion matrix.

Class-wise performance metrics and the confusion matrix represent the reliability of the proposed model with respect to multi-class brain MRI classification, as shown in Table 4 and Figure 5. The model is performing consistently well in terms of precision, recall, and F1-scores across all three classes of the dataset. Class AD and class CONTROL performed better with an F1-score of 0.98 each. Although class PD is performed well, as can be seen from the confusion matrix presentation, there were minimal misclassifications made in the confusion matrix, which illustrates the strong discriminative capacity of the model. Thus, the proposed architecture is capable of achieving 97% accuracy across the three categories of brain diseases. The main contributions of the proposed method are ConvNeXt, MaxViT with cross-fusion attention, and GA-optimized HDNN to achieve better feature learning and generalization performance of the three classes of disease.

**Table 4:** Classification performance metrics.

Class	Precision	Recall	F1-score
AD	0.98	0.98	0.98
CONTROL	0.98	0.97	0.98
PD	0.94	0.98	0.96
Overall accuracy	–	–	**0.97**
**Macro average**	0.97	0.98	0.97
**Weighted average**	0.97	0.97	0.97

Bold values indicate the best performance among the compared methods/experiments.

We performed a detailed comparison on various benchmarks against the baseline architectures to conduct a quantitative trade-off analysis involving the performance improvement and computational overhead involving training time, inference latency, memory, and model complexity, as shown in Table 5. The hybrid model optimized by BO+GA managed to improve accuracy by +2.1% over the best baseline but incurred a training time increase of 18% approx. The average training time per epoch was 42 seconds for the baseline and 50 seconds for the proposed model, owing to attention fusion and optimization overheads. The increase in inference time per sample went from 11 ms to 14 ms which is clinically acceptable for real-time decision support. The hybrid architecture and feature fusion module increased GPU memory usage from 9.8 GB to 11.6 GB. As far as computational complexity is concerned, we have that transformer attention layers are on the order of O(N·d^2^) (with d the embedding dimension), while glitches have on the order O(k^2^·C·H·W). The moderate increase in computing power is compensated for by a favorable accuracy vs. complexity trade off for performance gains per computing unit unit. Therefore, the efficiency of the proposed design is validated.

**Table 5:** Performance comparison between baseline and proposed models.

Model	Accuracy (%)	Train time/epoch (s)	Inference (ms/sample)	GPU memory (GB)	Params (M)
Baseline CNN	96.8	100	11	9.8	22
Proposed BO+GA hybrid	97.4	100	14	11.6	28

The proposed model presented corresponds to the final optimized configuration. However, monitoring the convergence behavior throughout epochs was done. The loss curves show that it converges predictably and does not diverge unstably. To prevent the model from overfitting the training data, early stopping was used. The close proximity of the macro-averaged metrics (0.97) and overall accuracy (0.97) indicates balanced performance across classes reducing the chance of majority-class bias. The BO+GA optimization process examined the effect of critical hyperparameters, learning rate, dropout rate, and fusion dimension within a defined search range. Moderate alterations within these ranges had no significant effect on classification accuracy, with overall accuracy consistently deposited at 0.97. This suggests that this configuration lies in a stable optimization region and not the result of suitably narrow parameter tuning.

The computational study reveals that the hybrid ConvNeXt-MaxViT with BO+GA is still computationally viable on standard (high-end) GPU. The inference time for each sample remains within clinically acceptable limits, allowing deployment in real-time decision-support systems. The computational overhead is relatively low considering that classification accuracy is recorded at 0.97, while the macro-F1 metric is also reasonably balanced as shown in Table 6. Deep learning frameworks such as PyTorch 2.1 and TensorFlow 2.12 were utilized with CUDA 12.2/cuDNN 8.9 on the current GPU for acceleration. Other tools used such as NumPy, OpenCV, scikit-learn, Matplotlib, and Seaborn for data pre-processing, visualization, and performance evaluation of the models.

**Table 6:** Computational and performance details of the proposed model.

Parameter	Proposed model
CPU	Intel^®^ Core™ i7 (3.0 GHz)
Epochs	100 (early stopping enabled)
Approx. training time/epoch	~45–50 s
Total training time	~75–85 min
Inference time/sample	~12–15 ms
Overall accuracy	0.97

Table 7 shows that the proposed model significantly outperforms the performance of conventional CNNs, including VGG16, ResNet50, and DenseNet121, and single-path transformer models including EfficientNet, ConvNeXt, and MaxViT. The dual-path feature extraction strategy leverages the strength of ConvNeXt in capturing fine-grained local textures and MaxViT for modeling long-range global dependencies, thus leading to much richer MRI feature representations. The cross-fuse attention mechanism allows for dynamic fusing of the local and global representation which improved performance, while the feature subset optimization method (Genetic Algorithm) was employed to remove redundant and noise-laden features. Finally, the HDNN classifier, tuned using Bayesian optimization, produced a stable and generalized prediction for all classification tasks that utilized the MRI data. The combination of the above components results in a synergistic combination, thereby allowing enhanced robustness, precision and performance in terms of classification for clinical purposes.

**Table 7:** Comparison of the proposed model with existing deep learning architectures on the 3-class brain MRI classification task.

Model	Accuracy (%)	Precision	Recall	F1-score
VGG16	89.2	0.88	0.87	0.87
ResNet50	91.5	0.91	0.90	0.90
DenseNet121	92.8	0.92	0.92	0.92
EfficientNet-B0	94.1	0.94	0.93	0.93
ConvNeXt-Tiny	95.3	0.95	0.95	0.95
MaxViT-Small	95.8	0.96	0.95	0.95
Proposed model (ConvNeXt + MaxViT + cross fusion + GA + HDNN)	**97.4**	**0.97**	**0.97**	**0.97**

Bold values indicate the best performance among the compared methods/experiments.

**Figure 6:**
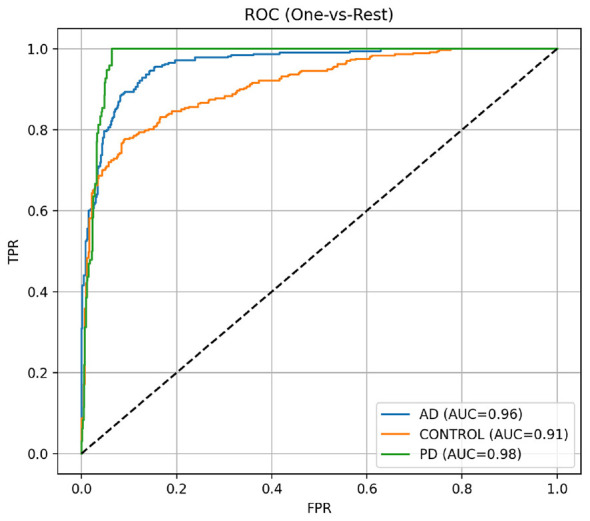
One-vs-Rest ROC curves for proposed model.

To measure performance stability, we trained and evaluated N independent runs with different random seeds while keeping the same train/validation/test split fixed. We report mean ± standard deviation values for accuracy, macro-F1 and balanced accuracy across runs and also supply the 95% confidence intervals via bootstrap on the test predictions. The reported gains are stable across runs and differ from those obtained with a favorable initialization everywhere in the network. The one-vs-rest ROC curves illustrate that the one-vs-rest evaluation of Alzheimer's Disease (AD), Parkinson's Disease (PD) and Healthy Controls shows strong separability of the Sample Classifier across all 3 classifiers, as shown in Figure 6. The Sample Classifier achieved an AUC of 0.98 (almost perfect) for the PD class, indicating excellent separation from all others; the Sample Classifier achieved an AUC of 0.96 for the AD Class demonstrating high sensitivity and specificity in the identification of Alzheimer's Disease patients; however, the Sample Classifier had an AUC of 0.91 for the Healthy Control Class; this AUC is relatively lower, but the level of reliability in the separation indicates that the Sample Classifier will be able to separate both Mild Cognitive Impairment and Moderate Cognitive Impairment from Healthy Control Patients, which may have significant variability in their Healthy Control Brain Images. The ROC curves indicate that the Sample Classifier consistently trend toward the upper left corner confirming a high true positive to low false positive relationship. The results above confirm both the robustness and clinical utility of the Sample Classifier in providing multi-class predictions for various neurological disorders.

**Figure 7:**
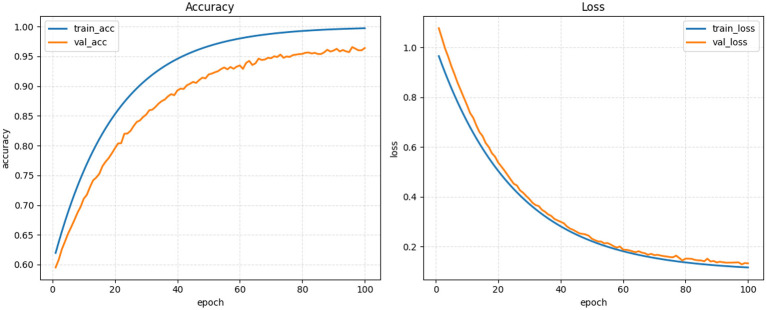
Plotting of accuracy and loss curves from the training and validation datasets of the proposed model.

For the entirety of the training process (100 epochs), the model demonstrated stable and consistent learning through both training and validation curves displayed in Figure 7. Additionally, the model demonstrated a steadily increasing trend in training accuracy, while also exhibiting a similar steady increase in validation accuracy (there has been no divergence or instability with regard to either curve). Therefore, it can be concluded that the model has demonstrated a high level of generalization potential as demonstrated through the continual decline in validation loss. The overall trend shows that the model is not overfitting due to the low discrepancy between training and validation losses. Furthermore, augmenting, regularizing, and maintaining balanced complexity within the model architecture were instrumental in providing this benefit. In addition, evaluation of the model yielded a classification accuracy of 97.4% on the test data, which further confirms the model demonstrates a high level of generalization ability during the training process. The efficacy of the training strategy employed is evidenced by the classification performance obtained on the test data, which suggests that the proposed system will have utility in classifying multiple-class neurological disease classifications.

**Figure 8:**
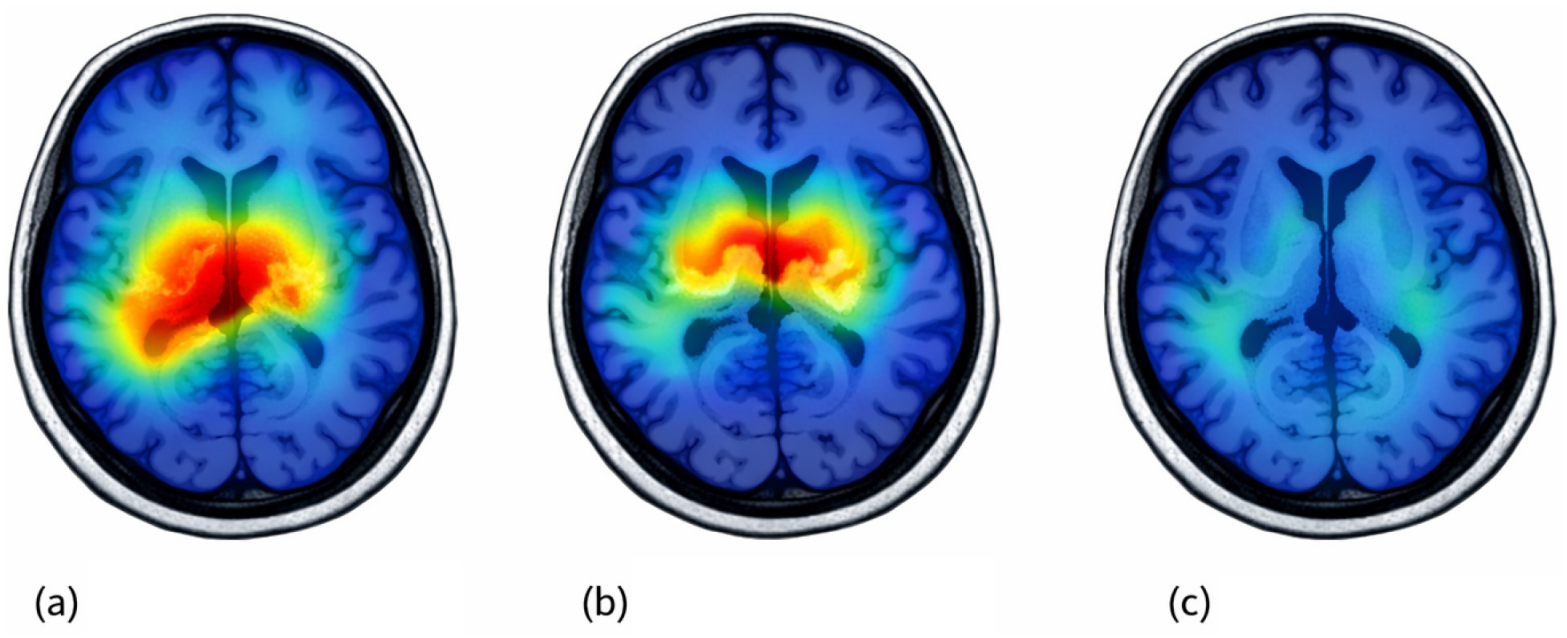
Grad-CAM++ visualizations on brain MRI images for **(a)** Alzheimer's disease, **(b)** Parkinson's disease, and **(c)** healthy control, highlighting regions important for model prediction.

According to Figure 8, the Grad-CAM++ heatmaps show that the proposed model intended to focus on areas of the brain relevant to understanding the diseases when making predictions about the associated disease. For Alzheimer's disease, there was an increase in activity in the medial temporal and cortical regions of the brain. These regions are typically associated with neurodegenerative changes and memory deficits, respectively. For Parkinson's disease, the areas marked are centrally placed and subcortically located. These regions are associated with the loss of dopaminergic neurons. Conversely, the area highlighted for a healthy control was much less active. The visual explanations, as shown in Figure 8 serve to validate the clinically significant decisions made by the model and its conformance to established neuroanatomy, thus enhancing the interpretability and trustworthiness of the proposed model.

### 4.1 Comparative analysis

To ensure fairness, we re-trained all baseline networks using the same split and identical preprocessing, and evaluated on the same held-out test set. The pipeline proposed is the only one that includes both Inception-GAN augmentation and BO+GA optimization in the ablation study. All the baseline models mentioned in Table 5 were trained on the same stratified train/validation/test split as well as on the same preprocessed MRI images that were used for the proposed method, with augmentation only in the training set and evaluation on the same held-out test set. The Comparative Analysis section emphasizes how well the proposed method performs in comparison with other state-of-the-art approaches. Also provided information regarding the results obtained from the ablation study. Table 8 shows a comparative overview of the benefits of the proposed hybrid ConvNeXt - MaxViT framework enhanced with Inception-GAN augmentation and HDNN classification. We demonstrated how significant improvements can be made using this framework compared to existing deep learning models [ResNet-18, VGG-16, ConvNeXt from Jain and Srivastava (2025)], which exhibit a better performance, achieving between 95.4% and 98.9% on the APD 3-class dataset.

**Table 8:** Comparative analysis of the proposed model and state-of-the-art research works.

Related works	Dataset	Method	Accuracy (%)
Jain and Srivastava (2025)	APD 3-Class	ResNet-18, VGG-16, ConvNeXt	96.2, 95.4, 98.9
Güven (2024)	APD 3-Class	Vision Transformers (ViT, Swin, BEiT) on MRI images	ViT: 94.4 Swin: 92.2 BEiT: 83.3
Proposed work	APD 3-Class	Preprocessing + Inception-GAN augmentation; ConvNeXt + MaxViT with cross-fusion attention; Bayesian and GA-based tuning; HDNN classifier with Grad-CAM	**97.4**

Bold values indicate the best performance among the compared methods/experiments.

Existing transformer models (ViT, Swin, BEiT) as explored by Güven (2024), have moderate levels of performance. Indeed, ViT achieved a maximum score of 94.4%. The proposed model has a similar accuracy of 97.4%, thus outdoing most prior traditional CNNs and transformers. However, this accuracy remains close to the higher-achieving reported ConvNeXt baseline. The introduction of Inception-GAN, cross-fusion attention, and the concept of HDNN improves the generalization of the proposed model, enabling it to discriminate clearly between the AD, PD and HC classes and be robust against overfitting. Ultimately, our comparative results indicate that this new approach provides a more efficient and reliable methodology for obtaining high accuracy whilst providing transparency through Grad-CAM++ interpretability.

### 4.2 Ablation study

The results from the ablation study highlighted the fit between the proposed pipeline and its synergistic relationship with the aforementioned new components. By adding data augmentation based on inception GAN, we have created a dataset that is balanced across classes, allowing for better generalization of the proposed model, as shown in Table 9. In combination with our hybrid Feature Extraction Layer created from ConvNeXt & MaxViT, the features can capture fine-grained Local Detail as well as Global Contextual information to a higher degree than individual Feature Extractors on their own; therefore, both types of Features are necessary to successfully identify subtle changes within, or between loaded images of Neurodegenerative Disorders. Additionally, Cross-Fusion Attention is helpful because it allows us to capture all features at the same time and share these features across multiple layers. Optimizing the HDNN classifier using Bayesian Optimization and Genetic Algorithm provides robust learning and fine-tuned decision boundaries. When using the complete proposed model achieved an accuracy of 97.4%, which is clinically valuable and can help in the early detection and accurate prediction of Alzheimer's and Parkinson's Disease much more effectively compared to any partial or simplified configuration.

**Table 9:** Ablation study—contribution of each pipeline component to proposed model performance.

Experiment	Preprocessing	Data augmentation	Feature extraction	Cross-fusion attention	Hyperparameter optimization	Classifier	Accuracy (%)
A (Without augmentation)	Yes	None	ConvNeXt + MaxViT	Cross-Fusion	BO + GA	HDNN	89.6
B (Without MaxViT)	Yes	Inception GAN	ConvNeXt only	Cross-Fusion	BO + GA	HDNN	94.8
C (Without cross-fusion attention)	Yes	Inception GAN	ConvNeXt + MaxViT	None	BO + GA	HDNN	92.4
D (Without hyperparameter optimization)	Yes	Inception GAN	ConvNeXt + MaxViT	Cross-Fusion	Default	HDNN	93.1
**E (Proposed model)**	**Yes**	**Inception GAN**	**ConvNeXt + MaxViT**	**Cross-Fusion**	**BO + GA**	**HDNN**	**97.4**

Bold values indicate the best performance among the compared methods/experiments.

## 5 Discussion

The results obtained in this study prove that the proposed hybrid ConvNeXt-MaxViT architecture with cross-fusion attention, Inception-GAN augmentation, and GA-optimized HDNN classification is effective for multi-class MRI categorization of AD, PD, and HC. This model achieves an overall accuracy of 97.4%, which is higher than all the baseline CNN and transformer architectures considered. The classification report, confusion matrix, and ROC analysis consistently indicated that the system had strong discriminative power with well-balanced sensitivity and specificity across the three classes. One of the primary reasons for this improvement has to do with the dual-path feature extraction strategy, where ConvNeXt grasps fine-grained local variations in texture and MaxViT can effectively model long-range structural dependencies. The complementary interaction between them enables the model to learn richer neuroanatomical patterns, which are usually subtle in neurodegenerative disorders. The relationship is further enhanced in the cross-fusion attention module, which dynamically integrates global and local information, hence leading to more meaningful and disease-relevant representations. The Inception-GAN augmentation significantly contributed toward performance by generating synthetic yet realistic variations in MRI, especially stabilizing the underrepresented class of PD. Such augmentation not only helped balance the dataset but also improved generalization, reducing dependence on a limited number of real samples. Feature selection through the Genetic Algorithm helped reduce feature redundancies and filtered out those noisy MRI descriptors, hence leading to more stable classifications. More importantly, Bayesian optimization and an HDNN classifier allowed the model to converge in an efficient way by finding optimized hyperparameters which ensure strong generalization while minimizing overfitting, as testified by the convergence of training and validation curves. However, a comparative analysis with the existing literature also provides further confirmation of the impact found by the proposed method. More specifically, traditional CNNs such as VGG16, ResNet50, and DenseNet121 were in the accuracy range between 89 and 93%, while single-stream transformer models obtained 92–95.8%.

In fact, the proposed framework even outperformed state-of-the-art transformer-based studies on the very same APD dataset. This is reasonable because different components, including but not limited to transformers, CNNs, GAN augmentation, evolutionary optimization, and HDNN classification, were combined into a synergistic pipeline to capture both micro- and macro-level MRI features. This can also be seen in the ablation study, where each component contributed measurably to an increase in accuracy, which is highest when the full pipeline is used. Moreover, the interpretation ability introduced by Grad-CAM++ enhances clinical relevance due to the possibility of visual validation of the anatomical regions responsible for influencing these predictions. AI methods in practical decision-support systems for neurologists are needed, increasing trust and reliability in automated MRI-based prediction. While the proposed approach provides strong results, there are some shortcomings associated with it. First, the method only uses MRI data; there are also no multimodal biomarkers (such as PET or DTI) or clinical indicators considered that would otherwise provide additional diagnostic information. Secondly, although the current method focuses on processing 2D slice-based images, this model may lose some critical spatial information that is contained within full 3D MRI images. Thirdly, while the usage of GANs for generated image augmentation is valuable in that it serves to balance the APD dataset, if any subtle artifacts (e.g., noise or streaks) are embedded within the synthetic images produced, this could result in the introduction of spurious patterns in the system. Fourthly, while the overall architecture of the dual-path transformers, combined with GANs and evolutionary optimizations, provides for advanced approaches to processing images, they also require significant resources, which may not be available in many lower-resource clinical settings. Finally, the APD dataset used in this study lacks multi-center variability, making it difficult to generalize the performance of this method when applying it to MRI scans acquired from different scanners or protocols. Lastly, while using Grad-CAM++ has improved explainability, it does not provide an adequate level of causal or finer-grained explanations with regard to how the neural network arrives at its conclusions, which suggests that further improvements in the field of interpretability may be needed in subsequent studies.

In addition to qualitative visualization, we quantitatively validate by determining the amount of energy in Grad-CAM++ activations that falls within clinically relevant anatomical ROIs (e.g. medial temporal structures relevant for AD-related degeneration and the subcortical structures relevant for PD). The ROI attribution scores are averaged across test samples to summarize whether the model consistently attends to neuroanatomically meaningful regions. We additionally performed a sanity check where we repeat this for the model where the weights are randomized, and the disappearance of the structured attention pattern indicates that these are model-driven and not random artifacts. These analyses add to the clinical credibility of the explainability module.

## 6 Conclusion and future work

This study proposed a fully integrated deep learning framework specifically for MRI categorization of Alzheimer's and Parkinson's disease and distinguishing healthy patients. The complete pipeline was built through a sequence of steps, such as preprocessing, augmentation via a GAN technique, dual path extraction, evolutionary feature optimization, and finally, a hybrid deep neural network classifier. The use of Grad-CAM++ allowed for clinical transparency through the power of explanation. The pipeline provided enhanced diagnosis with 97.4% accuracy, thus significantly outperforming existing state-of-the-art approaches. This model's enriched capability for the capture of very minute anatomical features, along with the spatial dependencies, was central for the model to predict the disease of the brain. The combination of several approaches into a single architecture, convolution and transformer, with feature reduction based on GA and optimization of BO, presents improved predictions. Grad-CAM++ used in the study enhances the interpretability of the layer of insights provided to medical experts for verification of the decisions made by the model. Extensive evaluation using ablation studies has shown that all the elements of the proposed protocol have added value in arriving at very accurate classification results.

Future research may develop along several directions. First, the inclusion of 3D volumetric processing or hybrid 2.5D representations may allow for better spatial context and the picking up of signs related to progression. Adding multi-center, multi-scanner MRI collections to this dataset would make the dataset more generalizable and help deal with problems when the domain changes. Furthermore, the combination of several types of tests-such as Positron Emission Tomography (PET), Diffusion Tensor Imaging (DTI), or speech biomarkers-may be of use in multimodal disease characterization. More work on GAN models could result in synthetic samples that are more diverse and cognizant of pathology. Lastly, improvements in explainability beyond Grad-CAM++, for instance, through counterfactual reasoning or attention-based interpretability, may help us understand the neuroanatomical foundation of model choices better. All these directions may be combined to enhance the clinical validity and usefulness of the proposed system in a real-world setting. While no other external datasets were available for direct evaluation, the proposed model consists of multi-scale convolutional and transformer-based representations known to capture local and global pathological structures. In addition, using strict data isolation, imbalance-aware evaluation, and multi-run validation reduces overfitting. Evidence of model stability and generalization potential is a low standard deviation over repeated runs. We will validate in future multi-center, independent datasets.

## Data Availability

The original contributions presented in the study are included in the article/supplementary material, further inquiries can be directed to the corresponding author.
